# An exceptional finding in an explanted liver: a case report of cirrhotomimetic hepatocellular carcinoma

**DOI:** 10.3389/fgstr.2023.1181037

**Published:** 2023-05-24

**Authors:** Elizabeth S. Aby, Susan M. Lou, Khalid Amin, Thomas M. Leventhal

**Affiliations:** ^1^ Division of Gastroenterology, Hepatology, and Nutrition, University of Minnesota, Minneapolis, MN, United States; ^2^ Department of Laboratory Medicine and Pathology, University of Minnesota, Minneapolis, MN, United States

**Keywords:** hepatocellular carcinoma, cirrhosis, liver transplantation, malignancy, case report, liver cancer

## Abstract

We present a case of cirrhotomimetic hepatocellular carcinoma (HCC) diagnosed on an explant following a liver transplantation (LT). The pre-LT computerized tomography (CT) scan demonstrated a nodular, cirrhotic-appearing liver; there was no evidence of lesions consistent with HCC. The level of serum alpha fetoprotein (AFP) 1 month pre LT was 4 ng/dL. Following LT, the patient underwent surveillance for HCC. Eight months post LT, he was noted to have lytic osseous lesions in his sternum and T10 vertebral body. Biopsies of these lesions demonstrated metastatic poorly differentiated carcinoma, which was concerning for progression to metastatic HCC. It is important to spread awareness of cirrhotomimetic HCC as it often evades detection by current screening methods, and if patients are inadvertently transplanted with a liver with cirrhotomimetic HCC, this can have significant consequences downstream. A multidisciplinary team approach is critical to ensure early detection of any recurrence and timely treatment.

## Introduction

Hepatocellular carcinoma (HCC) is the most common form of liver cancer and is the third leading cause of cancer-related deaths worldwide (1). Recent epidemiological studies suggest that the incidence rates for HCC in the United States have doubled in the past two decades with ongoing increases anticipated given the increasing prevalence of non-alcoholic fatty liver disease (2). Not only is the incidence expected to increase, but the number of annual HCC-related deaths has also doubled between 1999 and 2016 (3). Liver transplantation (LT) for certain patients with HCC offers excellent long-term survival. There is growing evidence that suggests that tumor size and number are just two of many factors that predict LT outcomes.

Most cases of HCC are diagnosed radiographically through the Liver Imaging Reporting and Data System (LI-RADS) system (endorsed by the American Association for the Study of Liver Diseases), which determines the likelihood of HCC through a combination of features including arterial phase hyperenhancement, enhancing capsule, delayed washout, and threshold growth (4). However, in some instances radiographic imaging fails to detect HCC. We present a rare variant of HCC, known as diffuse cirrhosis-like or cirrhotomimetic HCC, which mimics cirrhosis or nodular liver with no distinct mass. Although a rare condition, the recognition of cirrhotomimetic HCC is important given that it often evades pre-transplant detection and, if inadvertently transplanted, can have significant consequences downstream.

## Case description

A 62-year-old man with decompensated cirrhosis due to non-alcoholic steatohepatitis complicated by hepatic encephalopathy presented for a LT. His medical history included gastric antral vascular ectasia, hypothyroidism, transcatheter aortic valve replacement, diastolic heart failure, and type II diabetes.

Laboratory testing pre transplant was notable for a hemoglobin level of 7.1 g/dL, a sodium level of 143 mmol/L, a serum creatinine level of 3.03 mg/dL, a total bilirubin level of 13.2 mg/dL, an aspartate aminotransferase (AST) level of 178 U/L, an alanine aminotransferase (ALT) of 51 U/L, and an international normalized ratio (INR) of 1.92. The alpha-fetoprotein level (AFP) 1 month pre transplantation was 4 ng/dL, which is within normal limits. The alpha fetoprotein L3 (AFP L3) level was elevated at 36.8% (normal range 0%–9.9%). CT of the abdomen pelvis with IV contrast 4 months pre transplant showed a lobulated contour to a cirrhotic liver with splenomegaly, without evidence of lesions consistent with HCC. The portal vein was patent.

His family history was notable for hemochromatosis and HCC in his father. He underwent genetic testing for hemochromatosis, which was negative.

## Diagnostic assessment

**Figure 1:**
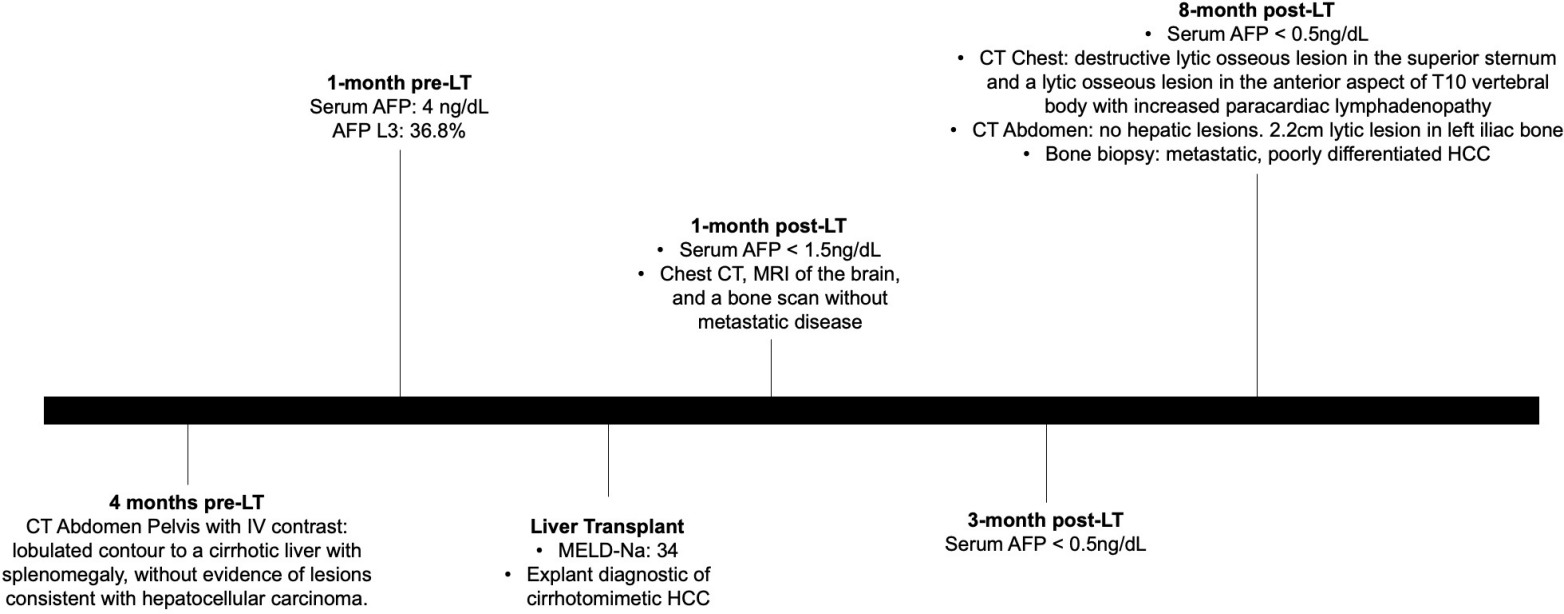
Timeline of events for the diagnosis and management of cirrhotomimetic hepatocellular carcinoma.

**Figure 2:**
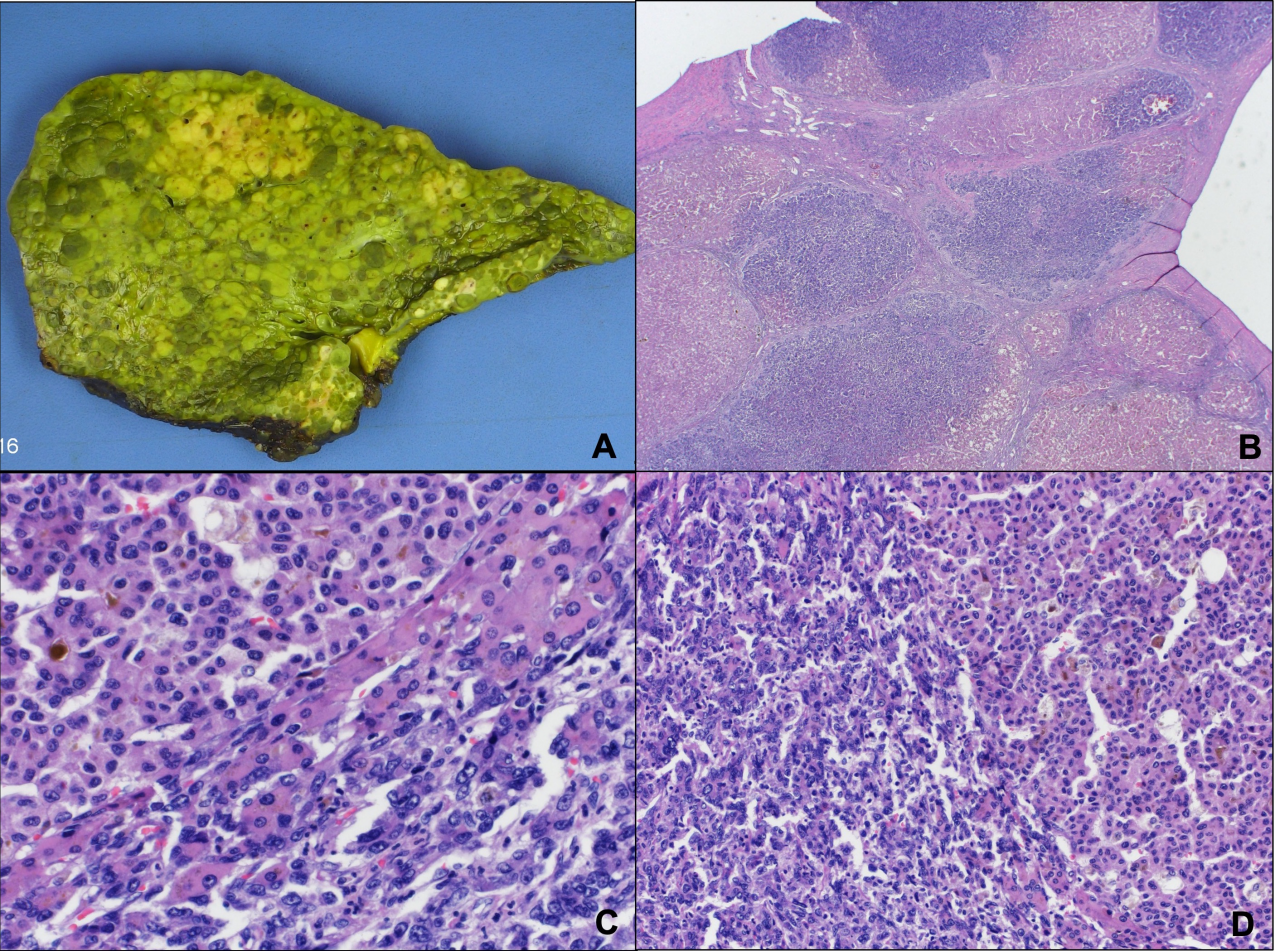
Multifocal, poorly differentiated hepatocellular carcinoma that involves the entire liver parenchyma, described as cirrhotomimetic. **(A)** The pathology demonstrated a nodular surface without any dominant tumor nodules. **(B-D)** Histopathologic findings showed evidence of high-grade hepatocellular carcinoma with marked nuclear pleomorphism and a prominent acinar pattern.

The patient underwent a deceased donor LT with a model for end-stage liver disease (MELD-Na) score of 34 (**Figure 1**). The pathology of the explant is as shown (**Figures 2A–D**), which is diagnostic of cirrhotomimetic HCC.

The explanted liver pathology was notable for multifocal, poorly differentiated HCC, which involved the entire liver parenchyma. The gross pathology demonstrated a nodular surface without any dominant tumor nodules (**Figure 2A**). Histopathologic findings showed multiple cirrhotic-appearing nodules with HCC (**Figure 2B**). On high magnification, there was evidence of high-grade HCC, with marked nuclear pleomorphism next to low-grade HCC with a prominent acinar pattern (**Figures 2C, D**). The bile duct and major vascular branches were uninvolved by the carcinoma. The appearance was described as cirrhotomimetic.

His hospital course of treatment post transplantation was complicated by persistent encephalopathy and seizures (requiring the adjustment of his immunosuppression to cyclosporine), a subsegmental pulmonary embolism, an abdominal wound abscess, persistent ascites, and pancytopenia. Given the patient’s abdominal wound abscess, the mammalian target of rapamycin (mTOR) inhibitor use was not considered immediately post LT given the associated issues with wound healing complications (5, 6).

The patient’s serum AFP level 1-month post LT was < 1.5 ng/dL. The patient’s 3-month post-LT AFP level was < 0. 5 ng/dL. He underwent a chest CT, an MRI scan of the brain, and a bone scan 1 month post LT without any evidence of overt metastatic disease. He followed with oncology and after discussion among the multidisciplinary team a surveillance plan was devised, including an abdominal MRI, a chest CT, and an AFP test approximately every 3 months. Unfortunately, on a chest CT 8 months post transplantation, he had evidence of an expansile destructive lytic osseous lesion in the superior sternum as well as a lytic osseous lesion in the anterior aspect of the T10 vertebral body with increased paracardiac lymphadenopathy, which is suggestive of metastatic disease. CT abdomen with IV contrast 8 months post transplantation showed typical post-surgical changes following LT without any focal hepatic lesions but was notable for a 2.2-cm lytic lesion in the left iliac bone, which was concerning for metastasis. Bone biopsy was notable for metastatic poorly differentiated carcinoma, which was concerning for metastatic HCC. The patient was initially started on sorafenib, but the patient had progression of his HCC while on sorafenib; therefore, he was switched to lenvatinib. A PET scan 1 month prior to his death demonstrated multiple lytic skeletal metastases, but no focal liver lesions. Given his metastatic lytic lesions in his iliac bone, palliative radiation was initiated for pain control. The patient died 14 months post LT.

## Discussion

Cirrhotomimetic HCC is a rare variant of HCC that produces small cirrhosis-like nodules throughout the liver. Given that the variant presents with innumerable small nodules, it can blend into the nodularity of a cirrhotic liver, and, thus, notoriously remain clinically and radiographically undetected (7, 8). In a single-center study of 26 cases, most cases of extensive cirrhotomimetic HCC were undetected pre transplant; 26.9% of cirrhotomimetic variants of HCC were discovered incidentally on explant (4). Most patients with the cirrhotomimetic variant of HCC had normal to mildly elevated AFP levels pre transplant (7, 8). In the published literature, most patients had AFP levels below 20 ng/dL, with only rare cases being recorded at levels up to 252 ng/dL (7, 8). Unlike our case, most tumor nodules were either moderately or well differentiated.

In this case, the patient’s AFP level pre LT was within normal limits, whereas their levels of AFP L3 were elevated. There is emerging data regarding the use of levels of AFP L3 and des-gamma-carboxyprothrombin (DCP) in conjunction with AFP as diagnostic tools for HCC (9). A prospective study assessing the role of AFP L3 and DCP in the LT setting suggested that levels of AFP L3 ≥ 15% and DCP ≥ 7.5 ng/mL were superior to levels of AFP alone in predicting high-risk explant features (10). Further work is needed to determine the role of AFP L3 and DCP in screening for HCC and predicting outcomes post LT.

The most prevalent histologic feature associated with the cirrhotomimetic variant of HCC is pseudoglandular architecture, which is more often seen in those with the extensive subtype (7, 8). In Clayton et al.’s classification schema for cirrhotomimetic HCC, based on tumor extent and cellular histopathology, tumors with a clear cell pathology were mostly associated with favorable outcomes (7).

Because cirrhotomimetic HCC is challenging to detect clinically and radiographically, it is often incidentally found following LT. Given that this variant often evades detection pre LT, the prognosis for these patients following transplantation is unclear. Despite the extensive nature, cirrhotomimetic HCC typically has an unexpectedly favorable survival (7, 8). In one small cohort, 60% of patients had no recurrence at follow-up (11).

Cirrhotomimetic HCC is a rare variant of HCC, which presents as a cirrhotic or nodular-appearing liver without a classic discrete mass. Although most cases presented in the literature are moderately or well differentiated, our case had components of high-grade HCC with marked nuclear pleomorphism. In addition, our case presented with metastatic poorly differentiated carcinoma, which was concerning for metastatic HCC following LT, suggestive of a poor prognosis. This case adds to the growing literature and suggests that not all patients with cirrhotomimetic HCC have a favorable survival post transplantation.

It is important to spread awareness about this condition as it often evades current screening guidelines for HCC, and if patients are inadvertently transplanted with a liver with cirrhotomimetic HCC, then this can have significant consequences downstream. Following cirrhotomimetic HCC diagnosis post transplantation, there is no standardized protocol for surveillance imaging or laboratory studies; therefore, a multidisciplinary team including, but not limited to, hepatology, transplant surgery, oncology, radiology, and interventional radiology, is critical to ensure the early detection of any recurrence and timely treatment.

## Data availability statement

The original contributions presented in the study are included in the article/supplementary material. Further inquiries can be directed to the corresponding author.

## Ethics statement

Written informed consent was obtained from the individual(s) for the publication of any potentially identifiable images or data included in this article. Written informed consent was obtained from the patient for the publication of this case report. 

## Author contributions

KA collected and analyzed the data. EA and SL collected the data, analyzed the data, and drafted the manuscript. TL designed and critically revised the work. TL is the article guarantor. All authors contributed to the article and approved the submitted version.
